# Editorial: New insights and future prospects of atrial cardiomyopathy

**DOI:** 10.3389/fcvm.2023.1264911

**Published:** 2023-10-06

**Authors:** Yan-Guang Li

**Affiliations:** Department of Cardiology, Beijing Anzhen Hospital, Beijing, China

**Keywords:** atrial cardiomyopathy, atrial fibrillation, anticoagulation, diagnosis, biomarkers

**Editorial on the Research Topic** New insights and future prospects of atrial cardiomyopathy

The term atrial cardiomyopathy (ACM) has been proposed for decades and is used to describe the pathophysiological change of the atrium. The concept of ACM has evolved in the past two decades. According to the expert consensus provided in 2017 by EHRA/HRS/APHRS/SOLAECE, ACM was defined as “Any complex of structural, architectural, contractile or electrophysiological changes affecting the atria with the potential to produce clinically-relevant manifestations” (1). However, it is impractical to make a unified diagnosis of ACM in clinical practice. Further steps of making clinical therapeutic decisions merely based on ACM still lack of evidence. Therefore, the notion of ACM has not been widely used.

ACM has caused much attention recently, attributing to its intimate relationship with atrial arrhythmia, majorly atrial fibrillation (AF), stroke, and atrial remodeling. In the previous studies, a lack of temporal relationship between AF and stroke has evoked the hypothesis that AF is not the direct reason for thrombogenesis and stroke; while the consistent underlying ACM is the chief culprit of AF progression and stroke. Multiple aspects of ACM can induce AF and stroke, including structural and electrophysiological remodeling, and abnormal atrial function. Although in the early stage of AF, trigger factor plays an important role in AF initiation, the progression and maintenance of AF merits a morbid atrium, which includes atrial enlargement allowing the existence of more wavelets reentry, extended low-voltage substrate for increased autorhythmicity. ACM interplays with AF in the vicious cycle of “AF begets AF”. A prospective multicenter study including 800 patients with embolic stroke with undetermined sources (ESUS) have revealed that nearly half individuals could be identified as with ACM, defined by left atrium enlargement and frequent supraventricular extrasystoles (2).

From clinical perspective, with current understanding, much attention has been paid to assessing the existence and extent of ACM. Electrocardiogram is the most economical way. Increased PtfV1 and P wave duration are the simplest markers for left atrial enlargement and conduction anomaly. Cardiac magnetic resonance imaging can provide the most accurate information of atrial structure, substrate (late gadolinium enhanced magnetic resonance), and function (4-dimensional flow cardiovascular magnetic resonance (3). While echocardiography is the most widely used approach for assessing atrial remodeling. Left atrial diameter has been used as a standard parameter for predicting AF relapse after pulmonary vein catheter ablation.

In the present special Issue, four study groups have provided new insights with regard to ACM from clinical and experimental perspectives. For example, Dr. Huang et al. has provided a predictive model for assessing individual potential for AF persistency. This model has included multiple well-accepted markers for ACM, including duration of amplified P-wave (APWD), left atrial volume, low-voltage-area extent, and mean left atrial voltage. The model acquired modest predictive accuracy, suggesting that these ACM-specific markers have the role of assessing AF progression risk. In the ARCADIA (Atrial Cardiopathy and Antithrombotic Drugs In Prevention After Cryptogenic Stroke) study, N-Terminal pro-B-type natriuretic peptide (NT pro BNP) >250 pg/ml has been used as one of the definitions for ACM, indicating the role of biomarkers for assessing the pathophysiological process of ACM (4).

For therapeutic decision-making, evidence regarding the role of ACM is still scarce. The most important topic is whether ACM could be used for guiding stroke prevention in patients with and without AF, and those with ESUS. Although the CHA2DS2-VASc scoring scheme is the mostly accepted tool for assessing individual risk for stroke in AF patients, while in non-AF patients and those with low CHA2DS2-VASc score but extensive scarred left atrium, such as those with atrial stasis, the decision regarding oral anticoagulants is still problematic. The ARCADIA trial aimed to investigate whether oral anticoagulant compared with aspirin would prevent recurrent ischemic stroke in patients with ESUS and at least one marker of ACM (4). In the recent report from the ARCADIA trial in 2023 European Stroke Organisation Conference (ESOC), oral anticoagulation did not provide benefit for this group of patients. Possible reason for this result may derive from the following aspects: (1) Biomarkers for defining ACM does not reach the severity of atrial pathological changes; (2) During follow-up, some patients from the control group received oral anticoagulant for new discovered AF; (3) ACM does not cause thrombogenesis without the presence of AF.

## References

[1] Goette A Kalman JM Aguinaga L Akar J Cabrera JA Chen SA EHRA/HRS/APHRS/SOLAECE expert consensus on atrial cardiomyopathies: definition, characterization, and clinical implication. Europace. (2016) 18:1455–90. 10.1093/europace/euw161 27402624 PMC6392440

[2] Ntaios G Perlepe K Lambrou D Sirimarco G Strambo D Eskandari A Prevalence and overlap of potential embolic sources in patients with embolic stroke of undetermined source. JAHA. (2019) 8:e012858. 10.1161/JAHA.119.012858 31364451 PMC6761628

[3] Spartera M Pessoa-Amorim G Stracquadanio A Von Ende A Fletcher A Manley P Left atrial 4D flow cardiovascular magnetic resonance: a reproducibility study in sinus rhythm and atrial fibrillation. J Cardiovasc Magn Reson. (2021) 23:29. 10.1186/s12968-021-00729-0 33745457 PMC7983287

[4] Huang T Nairn D Chen J Mueller-Edenborn B Pilia N Mayer L The AtRial cardiopathy and antithrombotic drugs in prevention after cryptogenic stroke randomized trial: rationale and methods. Int J Stroke. (2019) 14:207–14. 10.1177/1747493018799981 30196789 PMC6645380

